# Coastal Areas Division and Coverage with Multiple UAVs for Remote Sensing

**DOI:** 10.3390/s17040808

**Published:** 2017-04-09

**Authors:** Fotios Balampanis, Iván Maza, Aníbal Ollero

**Affiliations:** Robotics, Vision and Control Group, Universidad de Sevilla, Avda. de los Descubrimientos s/n, 41092 Seville, Spain; imaza@us.es (I.M.); aollero@us.es (A.O)

**Keywords:** remote sensors, Unmanned Aerial Vehicles, area partition, cell decomposition

## Abstract

This paper tackles the problems of exact cell decomposition and partitioning of a coastal region for a team of heterogeneous Unmanned Aerial Vehicles (UAVs) with an approach that takes into account the field of view or sensing radius of the sensors on-board. An initial sensor-based exact cell decomposition of the area aids in the partitioning process, which is performed in two steps. In the first step, a growing regions algorithm performs an isotropic partitioning of the area based on the initial locations of the UAVs and their relative capabilities. Then, two novel algorithms are applied to compute an adjustment of this partitioning process, in order to solve deadlock situations that generate non-allocated regions and sub-areas above or below the relative capabilities of the UAVs. Finally, realistic simulations have been conducted for the evaluation of the proposed solution, and the obtained results show that these algorithms can compute valid and sound solutions in complex coastal region scenarios under different setups for the UAVs.

## 1. Introduction

The extensive interest in the use of Unmanned Aerial Vehicles (UAVs) has led a large scientific, commercial and amateur hobbyist community to actively contribute to a broad spectrum of activities and applications. Some of these applications imply the deployment of a distributed swarm of UAVs as a sensor network, with or without the presence of other types of unmanned vehicles or static sensors.

For coastal areas, the complex geographical attributes and the increasing interest for activities in or near remote off-shore locations have raised challenges for marine environment protection and for sustainable management. European countries have vast coasts and economic zones that go far into the Atlantic and Arctic oceans and are challenging to monitor and manage. In addition, the European Strategy for Marine and Maritime Research states the need to protect the vulnerable natural environment and marine resources in a sustainable manner. The use of UAVs in coastal areas can provide increased endurance and flexibility, whereas they can reduce the environmental impact, the risk for humans and the cost of operations.

The study presented in this paper has been carried out in the framework of MarineUAS (http://marineuas.eu), a European Union-funded doctoral program to strategically strengthen research training on Autonomous Unmanned Aerial Systems for Marine and Coastal Monitoring. In particular, this study tackles the problem of complex area partitioning for a team of heterogeneous UAVs and the associated sensor-driven cell decomposition based on their on-board sensing capabilities. The proposed solution is a combination of computational geometry algorithms along with graph search strategies, which manage to partition an area regardless of the number of UAVs or their relative capabilities. Each UAV sub-area is decomposed into a sum of sensor-projection -sized cells, and a coverage strategy is computed in parallel for each of the produced configuration spaces. The strategy has been implemented as a network of ROS nodes [1], where the initial partitioning process is executed on the ground station and the cell decomposition and coverage planning are computed on-board each UAV.

The rest of this paper is organized as follows: Section 2 provides a review of the literature, presenting the current state of the art on cell decomposition and partitioning strategies for multiple vehicles. Section 3 describes the problem statement and the assumptions considered in the paper, whereas Section 4 describes the model adopted for the on-board sensors. Section 5 presents the two-step approach developed for the cell decomposition and partition of a complex coastal area considering the capabilities of a team with multiple UAVs. Results from the simulations are presented in Section 6 including a strategy for the generation of spiral inward paths for coverage. Section 7 closes the paper with a discussion on the results and future steps.

## 2. Related Work

In the aforementioned context, the literature provides many studies for an autonomous sensor network to achieve the task of complete area coverage. In an extensive survey for online and offline decomposition and coverage path planning algorithms, the authors in [2] provide a categorization of the techniques in the literature, showing that grid decomposition strategies and algorithms are mostly used in coverage tasks.

Several partitioning algorithms and strategies can be found for distributing known or unknown areas for a team of autonomous vehicles. In [3], the task of a safe and uniform distribution of many robotic agents has been researched, by using a generalized Voronoi diagram, creating a Voronoi partitioning of a complex area. This study also creates an initial grid decomposition, which converges to the computed Voronoi partitioning. Even though this study does not consider coverage path planning, the use of growing functions along with a modified version of the Dijkstra algorithm manages to successfully partition a complex area for multiple robots, while maintaining safe and fair distances between them. In a recent work presented in [4], the authors provide an algorithm for fair area division and partition for a team of robots, with respect to their initial positions. Their solution produces promising results regarding the algorithm’s computational complexity and guarantees full area coverage without backtracking paths. While their approach is feasible and manages to successfully tackle the problems of fair partition and coverage path planning, the strategy accounts only for the fair division case and fixed cell sizes, while in some cases, the sub-areas produced do not have a uniform geometric distribution.

Regarding multi-robot task allocation and distribution, the authors in [5] provide an extensive literature review and propose a novel taxonomy called iTax. This taxonomy categorizes the problems by complexity, where the first category includes problems that can be solved linearly, whereas the other three include NP-hard problems. The problem in our study is a general multi-robot task allocation problem, belonging in the latter category of the taxonomy, that of Complex Dependencies (CD). The ability to gradually reduce the complexity of the problem and to reach a lower level of complexity in each step can be exploited in the same manner that it is analyzed in the taxonomy.

In [6], the authors provide an optimal decomposition and path planning solution using an Integer Linear Programming (ILP) solver, by taking into account a camera-sized grid decomposition. Their solution manages to obtain the desired optical samples, although they do not provide the computational time needed for the ILP to find a solution. In the same context, the authors in [7] use an enhanced exact cellular decomposition method for an area and provide a coverage path consistent with the on-board camera of the UAV. Although their solution manages to produce smooth paths with minimal turns, their algorithms are tested only over convex polygon areas. The work presented in [8] deals with the same problem of area coverage for photogrammetric sensing. The authors include energy, speed and image resolution constraints in their proposed algorithms, such as an energy fail-safe mechanism for the safe return to the landing point. However, the provided solution and experiments do not account for complex, non-concave polygonal areas.

Regarding coverage algorithms for a single or a team of vehicles in a known area, the authors in [9] address the problem by creating an evaluation framework of path length, visiting period and balance workload metrics. In order to solve the problem, they generate a point cloud in which each point serves as guard in the art gallery problem, trying to maximize the visibility. The collection of these points, along with a Constrained Delaunay Triangulation (CDT) of the area, produces a graph where these points are the nodes. Thus, the final point cloud serves as a waypoint list, and coverage is achieved by using cluster-based or cyclic coverage methods. The work presented in [10] decomposes an area by using a convex decomposition and produces parallel lines in the decomposed parts, which are used as straight line paths. The algorithm presented tries to minimize the total amount of turns and provides a complete coverage plan. This strategy produces promising results, but complete coverage is not always achieved. Moreover, in some cases, repeated coverage is performed in order for the vehicle to visit the initial position of the next decomposed region. In [11], the authors tackle the problem of complete coverage by trying to minimize the completion time for the robots. In their strategy, turns imply the decrease in speed and eventually the acceleration of the vehicles. In that manner, their algorithm tries also to minimize the number of turns. This work also uses a grid-like decomposition strategy, based on disks. Once again, the areas considered for the experimental setups are convex rectilinear polygons. Finally, the authors in [12], provide a 3D coverage path planning strategy for underwater robots, and the analysis of the probabilistic completeness of the sampling-based coverage algorithm is shown. In their study, an underwater vehicle equipped with a sonar, a Doppler Velocity Log (DVL) and a camera obtains a 3D model of the ship to be inspected, while building and smoothing a roadmap for coverage. While the results are impressive, the amount, weight and energy requirements of the sensors are not compatible with the payloads of small or medium-sized UAVs.

Then, in general, the algorithms for cell decomposition, area partition and coverage planning do not take into consideration complex area characteristics or do not assume different sensor capabilities for the UAVs. In this paper, an exact cell decomposition strategy is applied in a novel algorithmic approach for area partition in a multi-UAV coverage mission context for coastal areas.

## 3. Problem Statement, Assumptions and Metrics Considered

The following scenario will be considered in this paper: an extensive oil leakage has been reported on an underwater pipe near a populated coastal region *R*, with particular aerial restrictions due to reserved airspace, nearby airports and domestic regions. A team of UAVs can be used for remote sensing purposes in order to localize the leakage sites and the extent of the oil spill around the reported sightings. The UAVs are heterogeneous with different autonomy capabilities and different on-board sensors.

Let $U_{1},U_{2},\cdots ,U_{n}$ be the team of *n* UAVs with initial locations $p_{1},p_{2},\cdots ,p_{n}$. These locations are relevant to the whole procedure since they are the places of the initially-reported oil sightings, and they have the highest probability of finding the location of the actual pipe leakage. Moreover, the dispersion probability of the oil spill by these locations is decreasing uniformly in all directions on the sea. As the UAVs are heterogeneous, each UAV may be in charge of the coverage of different percentages of the whole area *R*. Then, let $Z_{i}$ be the surface in square meters to be covered by $U_{i}$.

Let us consider an exact decomposition of the shape of region *R* into a set $S=C_{j},j=1,\ldots ,M$ of cells regardless of its complexity or the existence of no-fly areas, without any cells being outside or partially inside *R*. This requirement is crucial since the safe integration of UAVs in non segregated airspace is a key requirement of Single Sky European Research (SESAR) (http://www.sesarju.eu/newsroom/all-news/sesar-takes-next-steps-rpas); an exact decomposition of an area and the avoidance of flight over residential, commercial or restricted areas are ways to mitigate critical damage in case of system failures. In addition, the size of the cells should be consistent with the field of view of the sensors on-board the UAVs, as will be discussed later in the paper. The cell decomposition allows one to discretize the space in order to treat the complex geometry of *R*.

The goal is to compute the geometry of a set of sub-areas $A=A_{i},i=1,\ldots ,n$ based on the cells $C_{j}$ taking into account the following metrics:

- The closeness of the cells within $A_{i}$ to the initial location $p_{i}$ of the UAV in charge of searching that sub-area should be minimized. This can be achieved by minimizing the sum of distances between each center of cell $c_{ij}$ from the set *S* and the initial locations $p_{i}$:
  (1) $$\min_{S}F\left(S\right)=\min_{S}\sum_{i=1}^{n}\sum_{j=1}^{M_{i}\left(S\right)}∥p_{i}-c_{ij}∥\;,$$
  where $M_{i}\left(S\right)$ is the number of cells of *S* inside $A_{i}$.
- The size of $A_{i}$ should be as close as possible to $Z_{i}$ for all of the UAVs. This can be achieved by minimizing the sum of differences:
  (2) $$\min_{S}G\left(S\right)=\min_{S}\sum_{i=1}^{n}\left|\sum_{j=1}^{M_{i}\left(S\right)}\mathrm{area}\left(C_{j}\right)-Z_{i}\right|\;,$$
  where $\mathrm{area}(C_{j})$ represents the area of the cells inside $A_{i}$.

The former metric that takes into consideration the probability of localization led us to design an algorithm (see Section 5) where each sub-area is generated by a uniform growth from the starting locations $p_{1},p_{2},\cdots ,p_{n}$. This process has another positive side effect: since this growing region process is performed in every direction, it creates “symmetric” areas that are suitable to be covered by energy-efficient spiral-like patterns [13].

**Figure 1:**
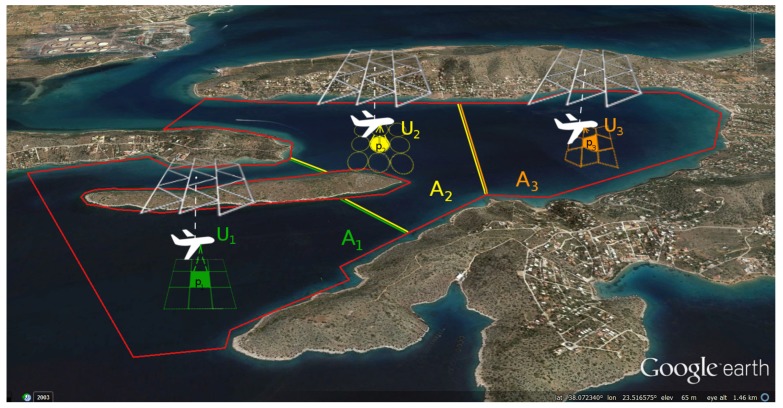
An example with three UAVs, each one with its allocated sub-area. The scheme is composed by two levels: the bottom layer shows the different on-board sensors’ field of view projection on the sea, whereas the upper shows the cell decomposition denoted as a triangular grid on top of each UAV. $U_{1}$, $U_{2}$ and $U_{3}$ denote the UAVs, and $A_{1}$, $A_{2}$ and $A_{3}$ denote the the sub-areas of the total region *R*, which is constrained by the red borders. The initial positions of the UAVs are $p_{1}$, $p_{2}$ and $p_{3}$.

In addition, $A_{i}$ by construction cannot be disjointed or intersected by another sub-area, neither by a no-fly zone. This restriction guarantees that the resulting sub-areas prevent the existence of overlapping coverage paths or collisions. In case additional safety requirements were present, it would be possible to define different flight altitudes for the UAVs in adjacent sub-areas. Figure 1 shows an example of a region *R* partitioned among three UAVs.

The next section describes the model considered for the on-board sensors since the cell decomposition should be consistent with the features of the sensors.

## 4. Model Considered for the On-Board Sensors

Regarding the use of on-board sensors, the literature mostly refers to cameras [14]. In those cases, by knowing the length, width and focal length of the camera, as well as the altitude from the sea, the shape of the projection of the Field of View (FoV) of the camera can be calculated based on the attitude of the UAV. This is not the case for point or side scan beam sensors, which have a wide width, but a really narrow length scanning profile. Then, in the following sections, the term FoV will be used to refer to the projection of the FoV of a generic camera on the sea.

**Figure 2:**
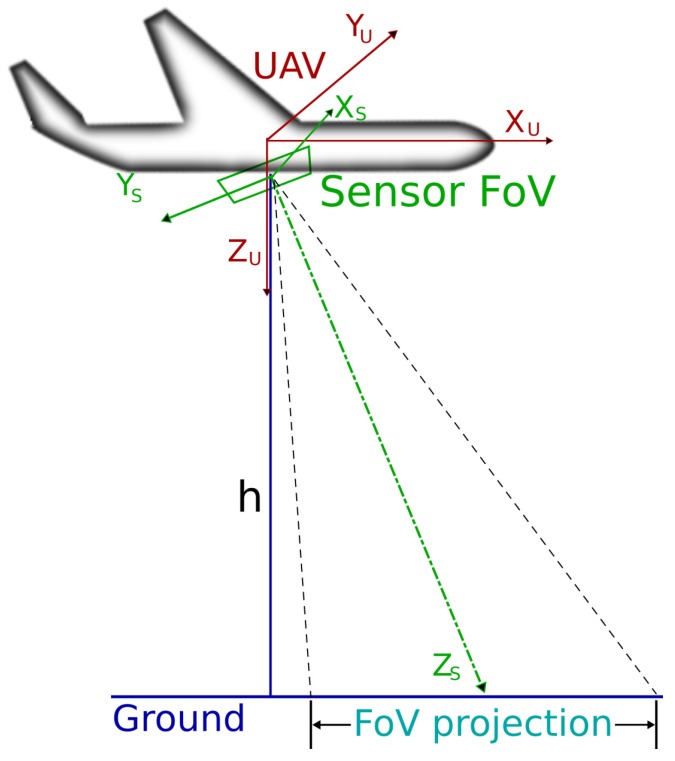
FoV projection calculation is relative to the coordinate frames of the system. For missions in coastal regions, the ground can be considered as flat.

Figure 2 represents the situation considered in this paper, with the on-board sensor inside a gimbal and pointing downwards a given angle with respect to the fuselage of the UAV. Thus, pitch and roll angles of the UAV with respect to the horizontal plane are not relevant to the FoV projection, since the gimbal compensates for these angles.

**Figure 3:**
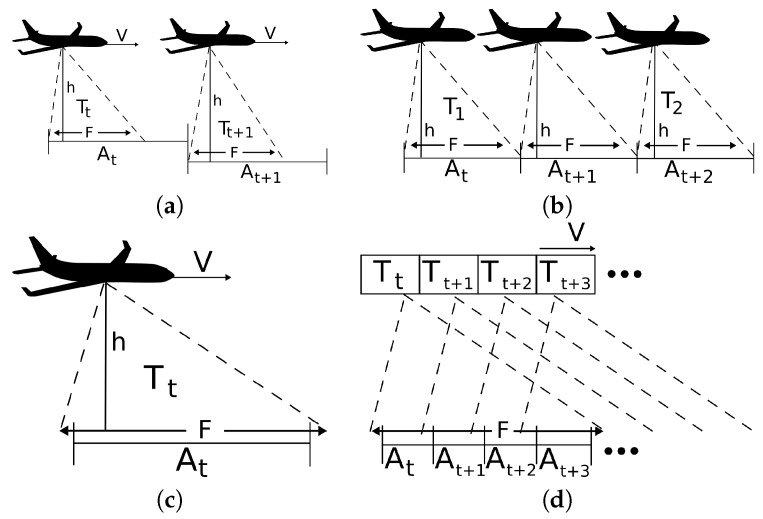
The appropriate cell decomposition is proportional to the velocity *V*, the sample rate *T* and the FoV projection footprint *F*. In (**a**), in every time step $t_{0},t_{1},\ldots ,t_{n}$, *F* is not large enough for the sensor to take a complete sample, whereas in (**b**), *T* is not fast enough to obtain a sample from each area. In both cases, the problem could be solved be either reducing the speed, increasing the sample rate if possible or increasing the altitude for increasing the projection of the FoV. In (**c**), the ideal solution in the limit is shown, whereas in (**d**), the most usual case of the same portion of the sea being present in many samples is presented.

The sample rate is another sensor characteristic to consider for the cell decomposition of a region. It is necessary to decompose the configuration space in a manner that a sensor can obtain at least one sample of each of the resulting cells in a unit of time (see Figure 3). Then, the projected footprint area *F* must guarantee that its size is proportional to the sample rate *T* and the UAV speed *V*. This can be achieved by either reducing speed or increasing altitude in order to grow the projection of the FoV. For most of the sensors, the sample rate is not an issue; however, this aspect has been pointed out for the sake of generality.

## 5. Area Decomposition and Partition in a Multi-UAV Context

This section describes the framework adopted by introducing the computational geometry tools for cell decomposition, as well as the algorithms for partitioning, based on the aforementioned considerations. These novel algorithms treat the segregated configuration spaces as topological graphs, allowing one to extract roadmaps for coverage planning after partitioning.

### 5.1. Exact Cell Decomposition

**Figure 4:**
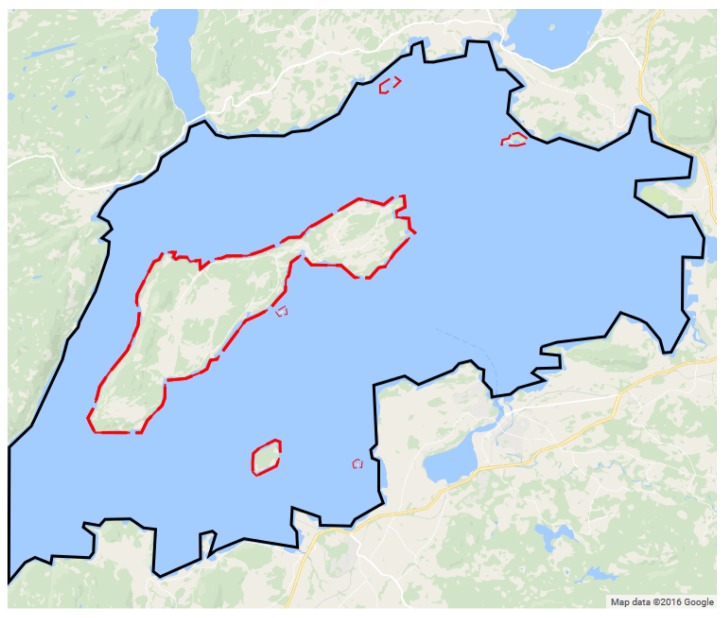
Trondheim fjord area (Norway) with Ytterøya island. The complex coastal area of interest is denoted by the black outer polygon, whereas the red dashed areas indicate regions that are no-fly zones.

In the example shown in Figure 1, the coastal area outlines a complex shape, similar to the one in Figure 4. In these cases, the surroundings are rarely the only area restriction, since several residential or industrial areas are no-fly zones inside this complex, non-convex polygon.

In order to decompose these kind of areas, the decomposition strategy in [15] has been followed, applying the Constrained Delaunay Triangulation (CDT [16]). This is performed by introducing forced edge constraints that define the area and the holes as part of the input. By using a Lloyd optimization [17] on the resulting triangulation, we manage to obtain even more homogeneous triangles as this optimization improves the angles of each cell, making each one of the triangle’s angles as close as possible to 60 degrees, depending on the selected iterations. By having more equilateral triangles, thus their angles closer to 60 degrees, a larger amount of area is covered in each step, and the overlapping during coverage is smaller.

The use of triangular cells for the decomposition is consistent with the complex shapes considered in the paper. The cells generated by the CDT are adapted to the shape of the borders, and this is very relevant since the center of the triangular cells is used for coverage planning. Hence, the paths computed based on this cell decomposition are initially consistent with the complex borders.

As has been previously mentioned in Section 4, each of the UAVs has a FoV projection, which guarantees that the speed along with the sample rate will manage to provide an adequate number of samples. This FoV size constraint is used as an input in the CDT method, being the maximum triangle side size. In order to guarantee that the FoV of each UAV will cover every triangular cell, regardless of its current orientation or sample rate, we need to provide a triangle side upper limit to be used as the CDT edge constraint. Then, if the centroids of the triangles are considered as waypoints, complete coverage could be achieved with the on-board sensor if a UAV follows that list of waypoints.

**Figure 5:**
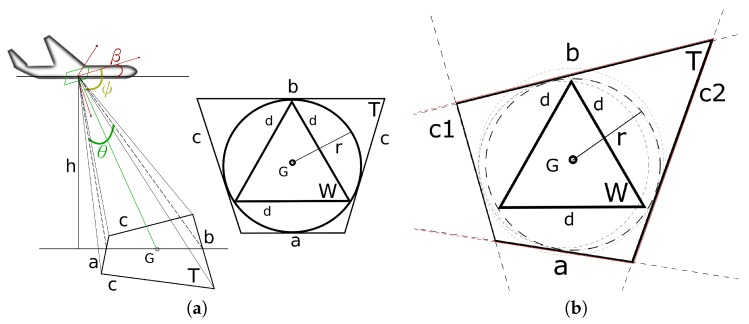
The FoV footprint, which is used in the test case of this paper, can be seen in (**a**) and forms a trapezoid *T*. The pitch angle β, along with the sensor’s view angle θ, the angle ψ that pitch β and the bisector of θ form and the altitude *h* from the ground are used for the calculation. The CDT constraint is defined by side *d* of the inscribed equilateral triangle *W*. Since *G* is the centroid of *T*, as well as *W*, the inscribed circle of *T* always coincides with the circumscribed circle of *W*. Then, the orientation of the two shapes is irrelevant. In addition, since *W* is the largest triangle that can be produced from the triangulation, any smaller triangles will always be inside the inscribed circle of *T*. In (**b**), the general calculation case is shown for no normal or tangential quadrilateral FoVs. In order to draw the maximum incircle of the quadrilateral, all four expanded triangles ($ac_{2}b$, $c_{2}bc_{1}$, $bc_{1}a$, $c_{1}ac_{2}$) must be drawn and their incircles found. Afterwards, the largest circle that is also an incircle of the quadrilateral is chosen, in this case the inscribed circle that is adjacent to sides *b*, $c_{2}$ and *a*, and it is used for extracting the CDT constraint.

Considering a generic camera as the on-board sensor, the FoV projection on the ground is a trapezoid *T*, as can be seen in Figure 5a. The projection shape depends on the pitch angle β of the UAV, the θ FoV angle of the sensor and their relative rotation matrices. The trapezoid has bases *a* and *b*, where *a* < *b*, and equal sides *c*. We inscribe a circle having the centroid *G* of the trapezoid as its center. Since we want the CDT to produce the minimum amount of homogeneous triangles, an equilateral triangle *W* is the largest triangle that can be inscribed inside the inscribed circle of the trapezoid. In that manner, we guarantee that a UAV will always cover each produced triangle since all of the triangles of that CDT are at most as large as *W* (see Figure 5).

**Figure 6:**
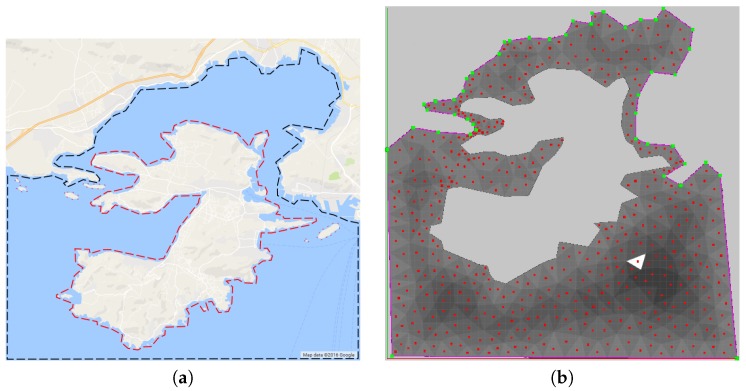
A CDT for a coastal region. In (**a**), the outer region constraints in black form a complex non-concave polygon. Several no-fly zones inside the constrained area are denoted in red. In (**b**) is depicted the CDT triangulation. The red dots represent the centroids of each cell. The shades of gray denote the Reverse Watershed Schema (RWS) formulation described later in Section 5.3.

From basic geometry, it is known that the radius of an inscribed circle in a trapezoid with bases of *a* and *b* is $r=\frac{1}{2}\sqrt{ab}$, and the side of an inscribed equilateral triangle in a circle is calculated as a chord of that circle and is given by $d=r\sqrt{3}$. Then, the upper limit side constraint of the CDT for that FoV can be easily computed. By using the aforementioned information, an initial cell decomposition is obtained based on FoV-sized triangles (see Figure 6).

### 5.2. Baseline Area Partitioning Algorithm

Let us consider an undirected graph G=(V,E), where the set *V* of vertices represents the triangular cells of the CDT and *E* is the set of edges such that there is an edge from $v_{i}$ to $v_{j}$ if the corresponding triangles are neighbors. Two triangular cells are neighbors if a UAV can move freely between them. This graph is intended to be used also to compute roadmaps for coverage path planning after the area partition is obtained. It should be mentioned that the CDT is computed based on the largest FoV among the available UAVs. Later, once the sub-areas $A_{i}$ are computed, another CDT is performed inside to fit the particular FoV of each UAV.

By treating the CDT as a graph, a baseline area partition algorithm can be designed based on two attributes for each vertex $v_{i}$ of the graph: $C(v_{i})$ as a unitary transition cost; and $A(v_{i})$ is the identifier of the UAV that will visit $v_{i}$. These attributes are computed as an isotropic cost attribution function by a step transition algorithm, starting from the initial position of each UAV, propagating towards the other UAVs or the borders of the area. Due to the fact that this algorithm expands in waves from each of the UAV and since each agent cannot overtake triangular cells of another agent and it progresses in a breadth-first manner [18], the strategy is called Antagonizing Wavefront Propagation (AWP).

This strategy is presented in Algorithm 1 and works as follows. Let us consider each of the initial positions of the UAVs as the root node of a tree; each root node is given an initial step cost of one. In every recursion step, each vertex that has an edge connected to the parent vertex is given that cost plus one. In addition, vertex $v_{i}$ gets the same $A(v_{j})$ attribute of its parent vertex $v_{j}$, propagating the identifier of the UAV in that way. In case the number of vertices for the $U_{k}$ UAV meets its autonomy limit, denoted by the total area $Z_{k}$ it should cover, the algorithm for that UAV stops. Please note that these steps are not performed if a triangular cell already has any of these attributes.
**Algorithm 1:** Antagonizing wavefront propagation algorithm that computes the baseline area partition. Q is a queue list managed as an FIFO by functions insert and getFirst. $v_{Ik}$: initial vertices/triangular cells for each UAV $U_{k}$,  $S_{v}$: area size of triangular cells *v*,  N(v): the set of neighbors of vertex *v*,  A(v): the UAV identifier allocated to triangular cell *v*,  $Z_{k}$: area coverage capability of UAV $U_{k}$ in square meters  $S_{vMin}$: area size of the smallest triangular cell in CDT  
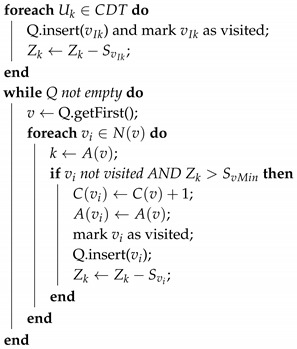


Since array *Q* is accessed once for every *i*-th cell, the *while* iteration has a complexity of O(n), where *n* is the number of vertices. The complexity of getting the first element is O(1); then, it inserts new elements according to the restrictions. The insertion in a stack has a complexity of O(1). Hence, the complexity of Algorithm 1 is O(n). The area partition computed is not sufficient for complex cases where a deadlock occurs after applying Algorithm 1. A further adjustment step is needed in order to assign regions where a deadlock happened, as will be described in Section 5.4.

### 5.3. Reverse Watershed Schema

By performing the previous step, each configuration space is either adjacent to another configuration space or to the borders of the whole region. In that manner, a second algorithm (see Algorithm 2) assigns to each vertex that already has an UAV identifier a unitary border-to-center cost attribute $D(v_{i})$ of proximity from the borders to the center of the configuration space. The triangular cells that are adjacent to a border with another configuration space or to the whole area are given a high $D(v_{i})$ cost and are considered as the root nodes of a tree. In each step of the algorithm, this cost is decreased and propagated to the adjacent triangular cells of these nodes. This function manages to create a border-to-center pattern resembling a watershed algorithm, and then, it is called the Reverse Watershed Schema (RWS) algorithm.
**Algorithm 2:** RWS algorithm for the generation of the border-to-center cost $D(v_{i})$ attribute. Q is a queue list managed as an FIFO by functions insert and getFirst. N(v): the set of neighbors of vertex/triangular cell *v*,  A(v): the UAV identifier for triangular cell *v*
 
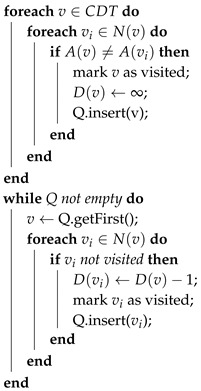


Here, we have to note that the complexity of this algorithm is similar to the previous one. The first loop has a complexity of O(n), O(n) for the initial *foreach* loop and O(1) for each insertion to the stack, since the second inner *foreach* has a maximum of three iterations. For the same reasons, the second *while* loop has also a O(n) complexity.

### 5.4. Adjustment Function for Deadlock Scenarios

**Figure 7:**
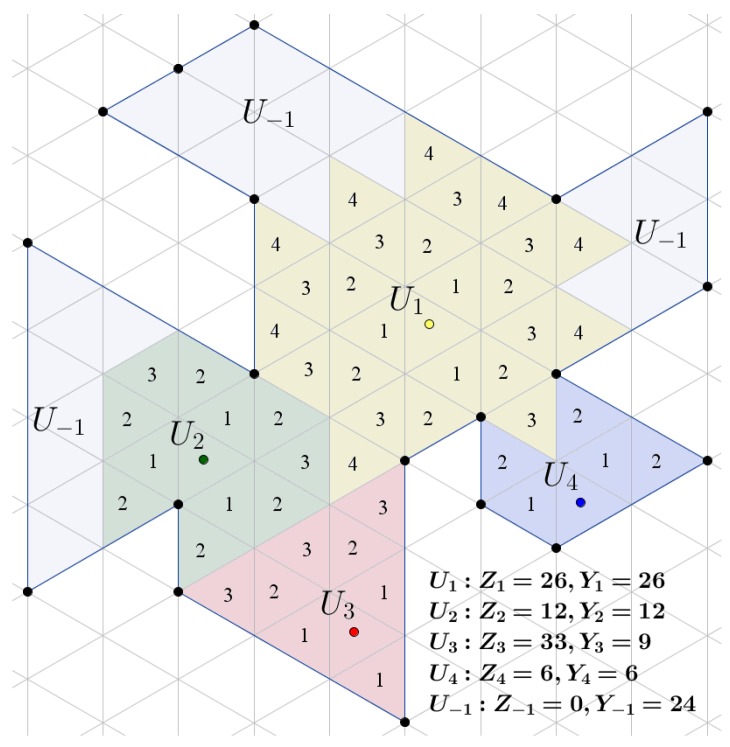
A deadlock scenario. Four UAVs $U_{1}$, $U_{2}$, $U_{3}$ and $U_{4}$ after the baseline partition Algorithm 1. UAVs $U_{1}$, $U_{2}$ and $U_{4}$ have met their autonomy capability of $Z_{k}$ by covering $Y_{k}$ area. Nevertheless, $U_{3}$ was not able to overtake any more area, being “blocked” by the other UAVs and the borders of the whole region. Colored areas indicate the configuration space of each UAV, while the numbers inside the cells indicate the isotropic cost, as has been assigned by Algorithm 1. The free or non-allocated areas belong to virtual UAV $U_{-1}$.

The previous baseline partitioning algorithm is able to perform well in most cases where the area is simple or where the initial positions of the UAVs are evenly distributed in the area. Nevertheless, it may lead to several deadlock scenarios as the growing sub-areas meet each other, as can be seen in the example shown in Figure 7. Hence, a Deadlock Handling (DLH) algorithm that adjusts the initial partitioning in the non-allocated areas is needed, by exchanging UAV identifiers or assigning UAVs to the empty areas. Two different approaches have been tested, by applying the two algorithms presented before.

**Figure 8:**
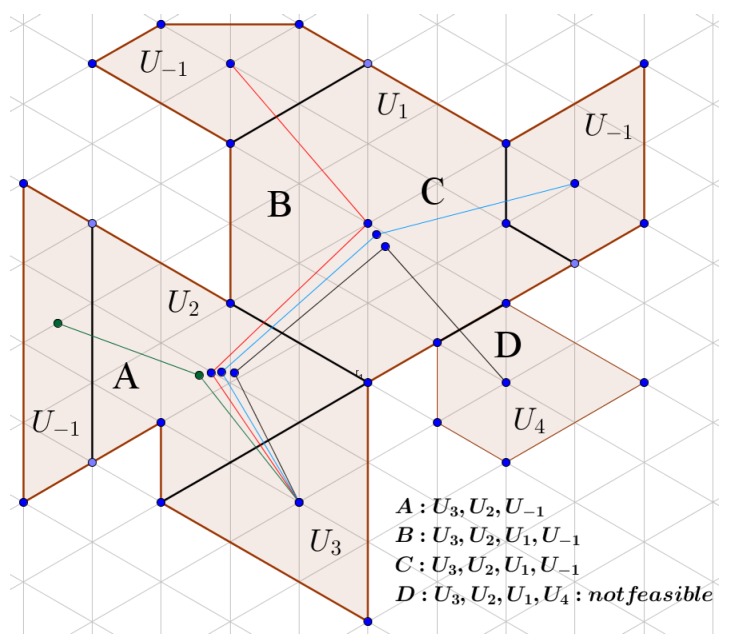
Transition sequence selection. After the initial partition process, $U_{1}$, $U_{2}$ and $U_{4}$ have met their sub-area size constraint and blocked the growth of $U_{3}$. As a result, three areas are not allocated ($U_{-1}$). The feasible transition sequences *A*(green), *B*(red) and *C*(blue) are used in order for $U_{3}$ to obtain the requested total area, by gradually exchanging cells in every pair of the sequence. Sequence *D*(black) does not lead to a partition that has an area shortfall, and thus, it is a not feasible sequence.

As was stated before, each UAV $U_{k}$ should cover an area of $Z_{k}$. In a test case, after the initial partitioning, let us consider that $Y_{k}\neq Z_{k}$ space has been allocated to $U_{k}$. In the deadlock scenarios (see Figure 7), there are areas that do not belong to any UAV. These areas are allocated to a virtual UAV $U_{-1}$ with area $Z_{-1}=0$. Let us consider a list $L_{U}$ that contains the results of $Z_{k}-Y_{k}$ for each UAV and the area size of the smallest triangle in the CDT $S_{vMin}$. Thus, each UAV can have an area surplus if $Z_{k}-Y_{k}>S_{vMin}$ or a shortfall if $Z_{k}-Y_{k}<S_{vMin}$. The latter case always happens in the deadlock scenarios for $U_{-1}$. In each recursion of Algorithm 3, a pair of UAVs, one having an area surplus and another with a shortfall, is chosen from the list in order to gradually exchange triangular cells between them to reach the desired area size. In order to do so, a feasible transition sequence must be found, as can be seen in Figure 8.
**Algorithm 3:** Multi-UAV partitioning Deadlock Handling (DLH) algorithm. Baseline partitioning is performed by Algorithm 1, whereas this method is for the sub-area size adjustment (if needed). Function getSurplusUAV(L) gets a UAV identifier from list *L* that has an area surplus, whereas function getShortfallUAV(L) gets the identifier of a UAV that has an area shortfall after Algorithm 1. Function findSequence finds a feasible transition sequence $P_{ij}$ between UAV $U_{i}$ and $U_{j}$, whereas the move function performs the transfer between triangular cells. $S_{vMin}$: area size of the smallest triangular cells in CDT  
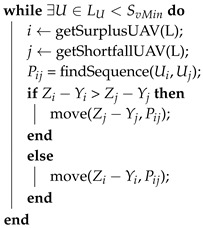


The complexity of this algorithm is calculated as $O(Un^{2})$ due to the *findSequence* function, which is actually a tree sort; *U* is the number of UAVs and *n* the number of cells. The complexity of the *move* functions is displayed below, in each of the following transposition algorithms.

Two algorithms called moveAWP and moveRWS have been implemented for the move function, which is used in Algorithm 3. In the former, the farthest vertex in the $A_{i}$ sub-area of UAV $U_{i}$ in sequence $P_{ij}$ is chosen, by using the information from Algorithm 1. This vertex has also to be adjacent to the second area in the transition sequence $P_{ij}$. Starting from that vertex, Algorithm 1 is applied again, overtaking the requested area size in the means of exchanging UAV identifiers between those triangular cells. Recursively, this operation is performed for every item of the sequence (see Algorithm 4). The complexity is O(n), where *n* is the number of areas that are in the transition sequence. Then, since Algorithm 1 is used for the transposition function, the whole complexity is $O(n*m^{2})$, where $m^{2}$ is the *findSequence* algorithm complexity.
**Algorithm 4:** MoveAWP algorithm. $C_{v}$ is the transition cost from the AWP algorithm (see Algorithm 1). Then, function $FindBiggestC_{v}\left(P\left[i\right],P\left[i+1\right]\right)$ finds the largest transition cost value triangular cell of UAV P[i] that is adjacent to UAV P[i+1] in the sequence. Then, function Awp takes as variables an initial cell *v*, the area size that needs to be exchanged and the UAV identifier that needs to be exchanged from. The growing function is similar to Algorithm 1. $P_{ij}$ the transition sequence between $U_{i}$ and $U_{j}$ for triangular cells exchange, treated as a list  $v_{init}$: initial triangular cell for identifier exchange  *S*: area size to be moved  
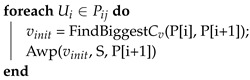


In the second approach, we apply the RWS algorithm in order to get a depth schema of the adjacent areas, as was described in Section 5.3. In each recursion of the algorithm (see Algorithm 5), the amount of triangular cells that are in the borders of the first pair of the transition sequence exchanges their UAV identifiers in order to change from $UAV_{P_{i}}$ to $UAV_{P_{i+1}}$. If the area of these border triangular cells sum up less than the requested area, then the area size and the total amount of border triangular cells exchange their UAV identifiers. If not, then only the triangular cells in the front (in the borders) exchange their UAV identifiers. This amount of triangular cells is then exchanged to the next UAV in the sequence and so on, maintaining the aforementioned restriction, until all of the requested area and associated triangular cells are transposed from the initial UAV in the sequence to the last.
**Algorithm 5:** MoveRWS algorithm. Function FindSequence finds a valid transition sequence, as can be seen in Figure 8. This function is also called before the initial recursion of the MoveRWS algorithm. Function ExchangeIdentifiers makes use of the information of the RWS algorithm (see Algorithm 2), and it exchanges agent identifiers on two adjacent configuration spaces, by exchanging the amount of triangular cells that have the lowest coverage cost, but are adjacent. It also propagates and extends this cost. Function RestOfSequence returns the remaining sequence for the specific P[i]→P[i+1] transition, in order to initially transfer only the amount of triangular cells that are adjacent between *i* and i+1 until the final $U_{j}$ UAV. In case this happens, the requested area has not been exchanged yet, so the algorithm runs recursively, and the last line takes a step back in sequence traversal. *S* area size to be moved  $S_{adj(kl)}$ the area size of adjacent triangular cells between UAV k and l  $P_{ij}$ the transition sequence between $U_{i}$ and $U_{j}$ for triangular cell exchange, treated as a list  
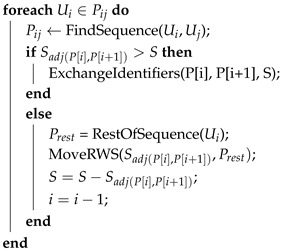


This algorithm’s complexity is $O(Un^{2})$ due to the use of the *findSequence* function, as has been described in Algorithm 3.

There are two main differences in these approaches. In the first approach, we have a wavefront pattern from a single triangular cell, whereas in the second, the exchange progresses as a kind of width sweep Morse function [2]. The second difference is that in the first approach, all of the triangular cells to be exchanged are the transposed UAV first, and in the second approach, only the amount of triangular cells that are in the adjacent borders are transposed in each step. In that manner, the triangular cells of the area are propagated respecting the total amount of cells that each UAV has each time, resolving overlapping UAV issues, as will be discussed in Section 6.

## 6. Simulation Results

The proposed algorithms are implemented in C++ using the CGAL library [19] for the constrained Delaunay triangulation and ROS (Robotic Operating System) [1] for the integration framework. In order to test the behavior of the UAVs, we have used a Software In The Loop (SITL) [20] simulation setup on a single computer, which is described later in Section 6.2.

**Figure 9:**
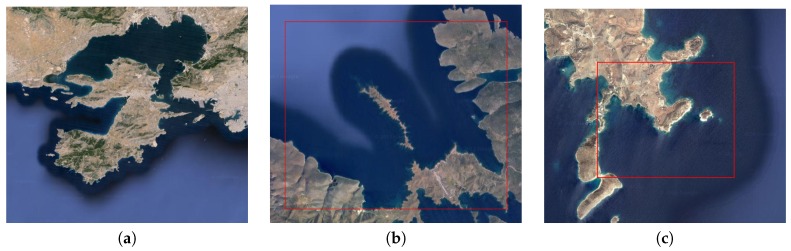
Selected areas for testing: (**a**) Salamina area having narrow passages and complex shapes in shores; (**b**) the Astipalea area is used for testing the suitability of the proposed algorithm; (**c**) Sxoinousa area used for coverage planning. The red square shows the region where the simulated flights occurred.

Three coastal areas in Greece have been selected for the experiments (Figure 9). The first is a broad and populated shore near the harbor of Piraeus, Salamina. The second and third are remote islands in the Aegean archipelago, Astipalea and Sxoinousa. The first area was used for evaluating and comparing the partitioning algorithms, the second for evaluating the proposed strategy in various setups, whereas the third was used for computing narrow coverage trajectories.

The coordinate frames chosen for the UAVs and on-board sensors and the software architecture used in the simulations are described in the following.

### 6.1. Coordinate Frames

**Figure 10:**
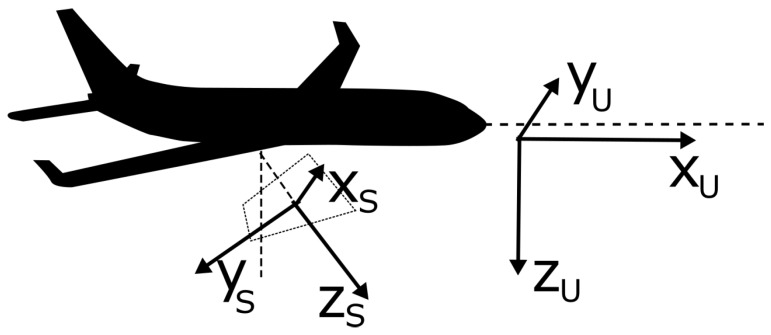
UAV and sensor coordinate frames and their relation. Depending on the roll (γ), pitch (β) and yaw (α) angles of the sensor with respect to the UAV fuselage, a rotation of the projected field of view occurs.

In the simulations, we have considered two reference frames, one for the UAV {U} and one for its on-board sensor {S}. For the UAV, the reference frame has its *x*-axis pointing forwards in relation with movement; the *y*-axis is given by the right-hand rule; while the *z*-axis points downwards. For the on-board sensor and its relation with the vehicle, the coordinate frame is shown in Figure 10.

The roll (γ), pitch (β) and yaw (α) angles are used to define the orientation of the sensor. In the γ=β=α=0 case, the $y_{S}$ sensor axis coincides with the $z_{U}$ axis of the UAV while the $z_{S}$ coincides with $x_{U}$. In that case, in order to translate a sensor vector to the UAV coordination frame, the rotation matrix used is:

(3) $$R_{U}^{S}\left(\alpha =0,\beta =0,\gamma =0\right)=\left[\begin{array}{ccc} 0 & 0 & 1\\ 1 & 0 & 0\\ 0 & 1 & 0 \end{array}\right].$$

Regarding the rotation movements along the three axes, the usual convention in aviation is used, where counterclockwise rotation movements of yaw, pitch and roll are considered. Yaw is the rotation of α about the *z*-axis; pitch is the rotation of β about the *y*-axis; and roll is the rotation of γ about the *x*-axis. These angles change the orientation of a given frame by applying the rotation matrix:

(4) $$\begin{array}{c} \begin{array}{cc} R_{U}^{S}=R\left(\alpha ,\beta ,\gamma \right)=R_{z}\left(\alpha \right)R_{y}\left(\beta \right)R_{x}\left(\gamma \right)=\\ =\left[\begin{array}{ccc} \cos\alpha \cos\beta & \cos\alpha \sin\beta \sin\gamma -\sin\alpha \cos\gamma & \cos\alpha \sin\beta \cos\gamma +\sin\alpha \sin\gamma \\ \sin\alpha \cos\beta & \sin\alpha \sin\beta \sin\gamma +\cos\alpha \cos\gamma & \sin\alpha \sin\beta \cos\gamma -\cos\alpha \sin\gamma \\ -\sin\beta & \cos\beta \sin\gamma & \cos\beta \cos\gamma \end{array}\right]. & \end{array} \end{array}$$

The on-board sensor orientation is derived by multiplying (3) by (4) and gives the full rotation matrix that allows one to transform a vector expressed in the on-board sensor frame to the UAV reference frame as:

(5) $$\begin{array}{c} \begin{array}{cc} R_{U}^{S}=R\left(\alpha ,\beta ,\gamma \right)=R_{z}\left(\alpha \right)R_{y}\left(\beta \right)R_{x}\left(\gamma \right)=\\ =\left[\begin{array}{ccc} -\sin\beta & \cos\beta \sin\gamma & \cos\beta \cos\gamma \\ \cos\alpha \cos\beta & \cos\alpha \sin\beta \sin\gamma -\sin\alpha \cos\gamma & \cos\alpha \sin\beta \cos\gamma +\sin\alpha \sin\gamma \\ \sin\alpha \cos\beta & \sin\alpha \sin\beta \sin\gamma +\cos\alpha \cos\gamma & \sin\alpha \sin\beta \cos\gamma -\cos\alpha \sin\gamma \end{array}\right]. & \end{array} \end{array}$$

However, as was mentioned in Section 4, pitch and roll angles of the UAV with respect to the horizontal plane are not relevant to the FoV projection, since we are considering a gimbal on board, which compensates for these angles.

### 6.2. Simulation Architecture and Configuration

**Figure 11:**
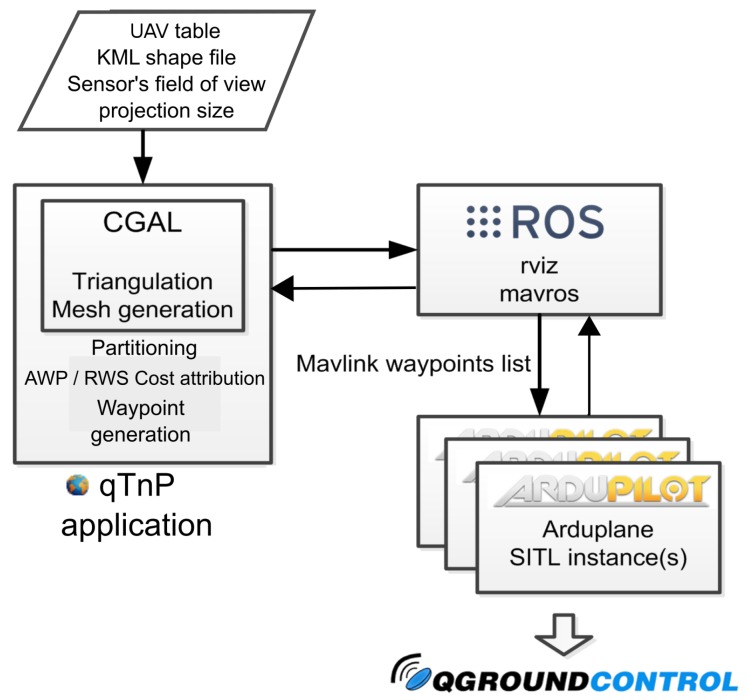
Software architecture with different libraries and components: the latest CGAL library (4.8.1) [19], ROS Indigo [1] components (the rviz package [21] for visualization and the mavros node [22] for the mavlink interface with the simulated UAV), an Arduplane instance [23] of the Ardupilot SITL [20], which uses the JSBSim flight dynamics model [24], and the qgroundcontrol control station [25].

The simulations have been performed on computers with an Intel Core i5-5200U@2.20-GHz CPU with 8 GB of RAM and the kUbuntu 14.04 distribution of the Linux OS. The software architecture adopted is shown in Figure 11.

**Figure 12:**
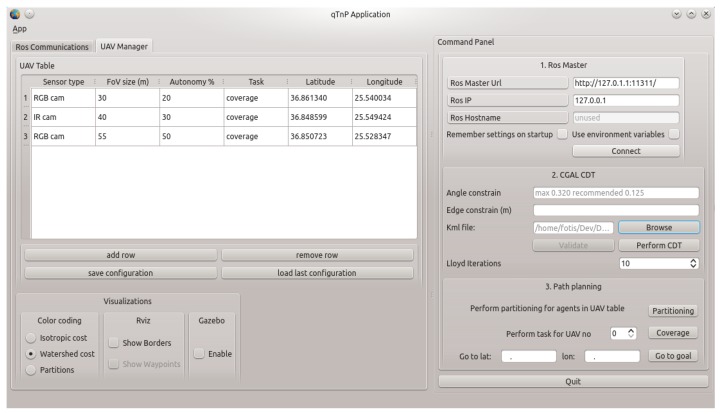
The qTnP main application. The first tab echoes the ROS communication messages and logs. The main "UAV Manager" tab of the application includes the UAV management table, indicating the sensor type, the cell (FoV) size, autonomy percentages and initial positions. It also includes the visualization options for rviz, showing the cost values of each of the proposed algorithms, visualizing the partitioned configuration space, showing the borders of each UAV and the produced waypoints for coverage. Finally, the command panel on the right includes connection settings, CDT-specific configuration, the KML file of the area, as well as several command buttons for the different stages of the experiments.

The main application is based on the Qt (https://www.qt.io/) cross-platform software development framework. The setup consists of a configuration window (Figure 12) where the number of the UAVs along with their attributes can be set. These attributes are the sensor type, the FoV size referring to the maximum triangular side size, as had been defined in Section 5, a percentage of the whole region to be used in the partition step, initial positions and tasks. The configuration application sets the type of visualization that will be performed in rviz: showing the borders of each sub-area, coloring it depending on different parameters and showing the produced waypoints for coverage. Regarding the CDT, its constraints of minimum angle and initial triangulation maximum edge can be also defined, and the user can define the area of interest by uploading a KML file, including obstacles. Finally, each step of the simulation can be performed separately; performing the triangulation, extracting the partition for each UAV based on its percentage of the total region and computing coverage waypoint plans for each UAV.

The implemented algorithms are part of an ROS node named qTnP (Qt Triangulation and Planning, Figure 12). This node performs all calculations and manages the communication with the rest of the ROS nodes of the configuration. Visualization of the mesh of the area, partitioned areas, cost attribution, waypoints and produced paths is handled by the rviz node, whereas the produced waypoint stacks are sent to mavros node. This node has a dual purpose. It maintains the connection with the simulated vehicles, sending waypoint list plans when the main application produces them. It also listens to the simulated UAVs, which report the mavros node on each cycle for their current position and telemetry data.

Regarding the UAV model used in the simulations and its on-board controller, the open source autopilot Ardupilot has been used. Its arduplane instance for fixed wing model aircraft has been combined with the JSBSim flight dynamics model simulator. In our setup, the system simulates the dynamics of the Rascal110 model airplane. The Arduplane controller used is the Pixhawk Flight Management System [26]. The behavior of the vehicle during the simulated flight, as well as the produced trajectories were monitored live using the open source ground station qgroundcontrol [25].

### 6.3. Partitioning Algorithms Comparison in Simulation

**Figure 13:**
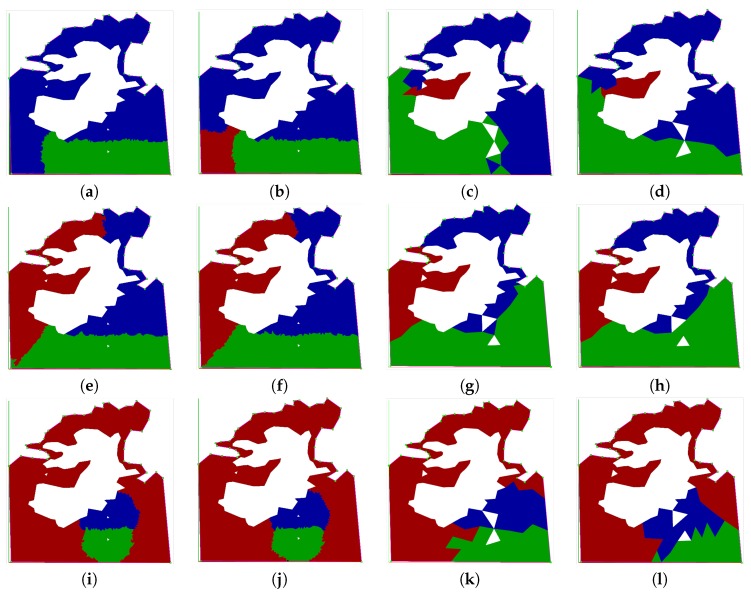
Partitioned area for three UAVs (indicated by the white cells) and visualized by using the ROS rviz node. Each row represents the results for different relative capabilities: the first row is the 10% (red), 60% (blue), 30% (green) case; the second row depicts the 33% (red), 33% (blue), 34% (green) case; whereas the last row shows the 80% (red), 10% (blue), 10% (green) case. In each row, each pair of images indicates the comparison of the two algorithms. (**a**,**b**) show how the MoveAWP and MoveRWS algorithms have performed with the small (250 m) FoV, whereas (**c**,**d**) show the results for the large (2 km) FoV case. (**a**) MoveAWP 250 m FoV; (**b**) MoveRWS 250 m FoV; (**c**) MoveAWP 2 km FoV; (**d**) MoveRWS 2 km FoV; (**e**) MoveAWP 250 m FoV; (**f**) MoveRWS 250 m FoV; (**g**) MoveAWP 2 km FoV; (**h**) MoveRWS 2 km FoV; (**i**) MoveAWP 250 m FoV; (**j**) MoveRWS 250 m FoV; (**k**) MoveAWP 2 km FoV; (**l**) MoveRWS 2 km FoV.

The partitioning strategies called MoveAWP and MoveRWS described in Section 5.4 have been compared. The former uses the transition cost of the AWP algorithm, and the latter applies the RWS algorithm for adjusting the baseline partition computed by Algorithm 1. In both cases, two FoV sizes have been used, in order to show the impact in the behavior of the algorithms of small and large values. The FoV size values in the simulations refer to the maximum triangular cell side, as has been defined in Section 5. Three test scenarios were simulated with different relative capabilities for the UAVs, and the results are shown in Figure 13.

The complexity of the area has managed to highlight some issues that were not evident for the majority of simple areas. The main problem occurs during cell exchange when the initial position of the UAV is close to the borders, because a sub-area could overtake the initial position of the UAV (see Figure 13a).

**Figure 14:**
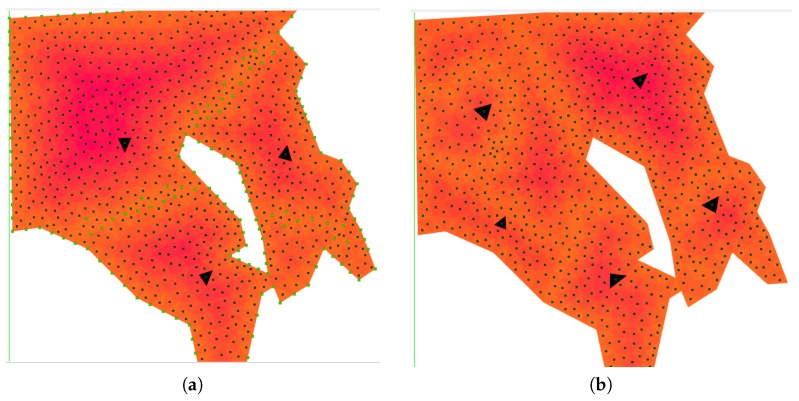
Area of Figure 9b selected for the comparison of the two partitioning algorithms. (**a**) is partitioned for three UAVs, whereas (**b**) for five UAVs. The depicted FoV size is 30 m in both cases. Both figures are computed with the deadlock moveRWS handling of Algorithm 5.

Additional simulations have been performed to measure the performance of the different algorithms with respect to the metrics *F* and *G* explained in Section 3. In particular, the simulation environment shown in the second area of Figure 9 has been used for the metric *F*. Some results are detailed in Figure 14 for three and five UAVs and a FoV size of 30 m. In general, simulations have been executed for three and five UAVs, with initial locations evenly or randomly distributed in the area and different FoV sizes. The results for the sum of distances between each center of the triangular cell inside a sub-area and the initial location of the UAV inside that sub-area (metric *F*) are shown in Table 1 and Table 2. In both cases, it can be seen that the moveRWS algorithm has a better performance than moveAWP, since the metric is lower.

**Figure 15:**
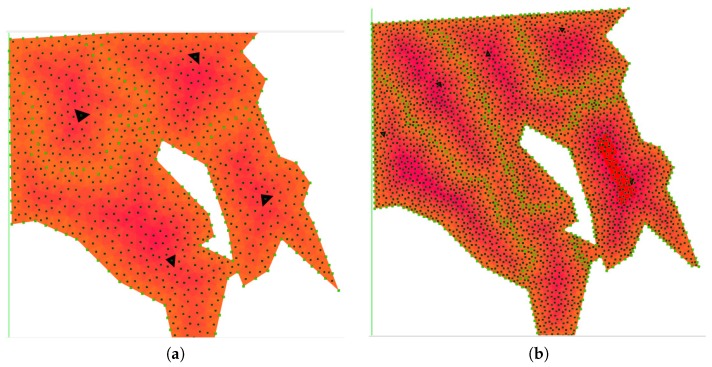
Area partition after applying the baseline and the deadlock moveRWS handling algorithm. (**a**) shows the area partitioned for four UAVs evenly distributed in the area and a FoV projection of 30 m. (**b**) shows the results for a FoV projection of 15 m and four UAVs randomly located. The black triangles depict the initial positions in all of the cases.

Regarding the other metric *G* considered in Section 3, simulations have been performed also in the second scenario of Figure 9 with three and six UAVs with different relative capabilities and initial locations evenly and randomly distributed (see Table 3 and Table 4, respectively). The goal is to compare the results computed with the baseline algorithm and the improvement achieved with the moveRWS deadlock handling algorithm, which had the better performance in the previous scenarios. Figure 15 shows the results for two particular setups with four UAVs.

**Figure 16:**
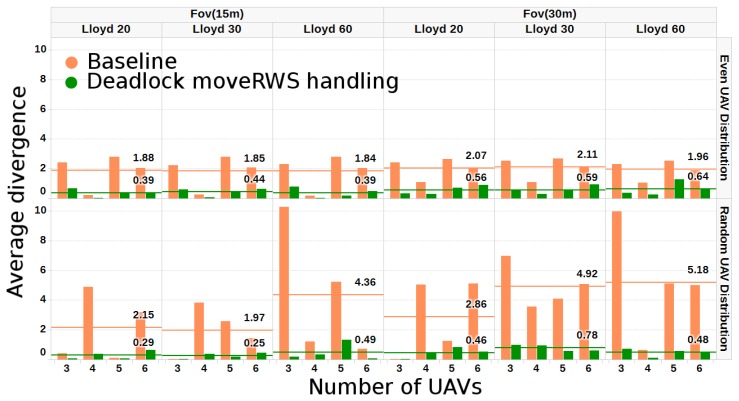
A graphical representation of Table 3 and Table 4. Average difference after the baseline algorithm and after the deadlock moveRWS treatment algorithm. As expected, random initial positioning of UAVs creates more often deadlock scenarios for the baseline algorithm. The algorithm has been tested for 3–6 UAVs, evenly or randomly distributed in the area. FoV projections of 15 and 30 m have been tested, and in each case, a different Lloyd iteration setting (20, 30 and 60) has been set. The horizontal lines show the average difference.

Finally, different numbers of Lloyd iterations on the resulting mesh have been tested, ranging between 20 and 60 iterations. In the simulations, different numbers of UAVs with even and random distributions for the initial locations (see Table 3 and Table 4, respectively), different relative capabilities and FoV projections have been used. The results show the suitability of the proposed solution, as the average difference from the targeted relative capability of the UAVs has not exceeded a value of 1% on average for the even distribution and 1.33% for the random distribution of the initial locations; Figure 16 shows this comparison of the average difference in the even and random distribution scenarios. Moreover, the algorithm manages to properly overcome deadlock scenarios as expected, as can be seen in the various setups of Table 4, where the initial baseline algorithm has up to 30% difference from the targeted relative capabilities of the UAVs.

### 6.4. Coverage Path Planning Simulation Results

**Figure 17:**
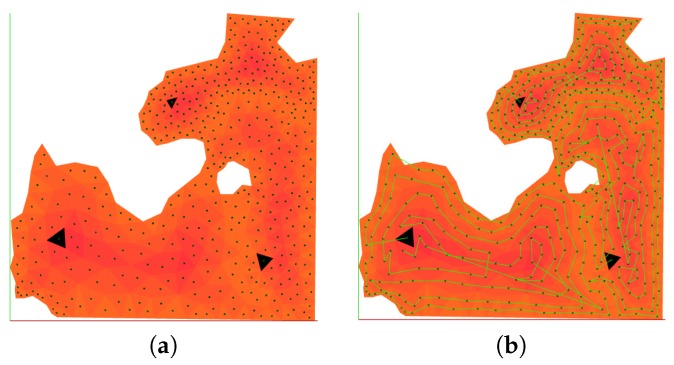
Area partitioning for three UAVs on the same location as in Figure 9c. White areas indicate the no-fly zones, whereas the black triangles show the initial positions of the UAVs. In (**a**), the FoV sized cell distribution is shown along with the centers of the triangles. Regarding waypoint generation for coverage, (**b**) shows the produced coverage paths for all of the UAVs. The different shades of orange indicate the border-to-center cost computed by Algorithm 2.

The framework presented in the previous sections, and in particular Algorithm 2, can be also applied to generate waypoint lists for the UAV to achieve complete coverage of a complex coastal sub-area. By using the border-to-center cost described in Section 5.3, inward spiral-like waypoint lists *W* can be generated. Algorithm 6 performs a selection of vertices by initiating from the vertex that has the highest D(v) cost and is closer to the starting position of the UAV. In every recursion, the closest adjacent cell $v_{j}$ that has the same cost ($D\left(v_{j}\right)=D\left(v\right)$) is inserted in the list. In case all of the selected vertices have the same cost, the algorithm reduces the visiting cost and chooses the cell that is closer to the previous step. It should be mentioned that the complexity of this algorithm is $O(n^{2})$.
**Algorithm 6:** Waypoint list computation for coverage. $D_{c}$ is an auxiliary variable with the current border-to-center cost in each step, whereas $v_{I_{k}}$ is the starting position of the UAV $U_{k}$. Function findClosest finds the closest vertex to the current one that has its same border-to-center cost. $CDT_{k}$ is the sub-CDT for UAV $U_{k}$. *W* is the produced waypoint list of vertices. $D_{c}\leftarrow \infty$;  v← findClosest($v_{I_{k}},D_{c}$);  *W*.insert(*v*);  
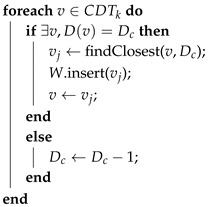


In order to show the coverage trajectories computed, tests have been performed in a particular sector of the area considered in Figure 9c. The area was partitioned into sub-areas respecting the different UAV capability considerations and coverage waypoint lists have been produced, as can be seen in Figure 17a, whereas Figure 17b shows the coverage waypoint trajectories for each UAV. Even though the number of turns is higher in comparison with a square grid decomposition strategy, the area was fully covered fulfilling the constraints considered in this paper. Table 5 shows the values of the parameters considered for the UAVs in this simulation.

**Figure 18:**
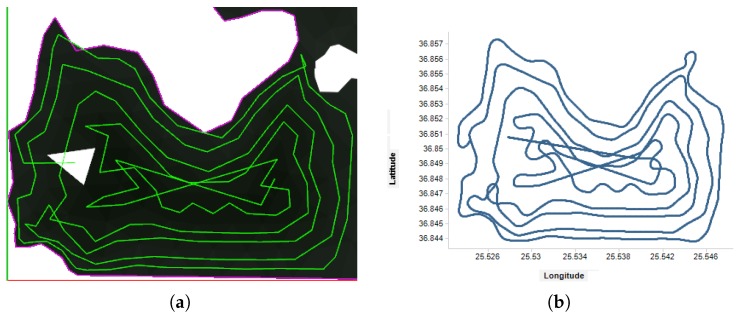

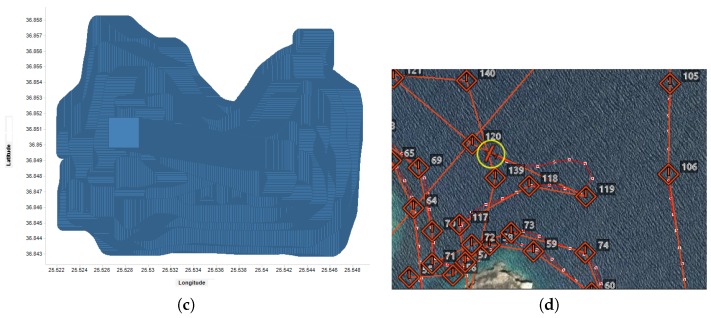
Coverage trajectory computed in the simulation. (**a**) shows a detailed view of the UAV 3 trajectory in Figure 17. Latitude and longitude information received during the simulated flight of that UAV is shown in (**b**,**c**) with the total sensor coverage considering a sensor working at a very slow rate of 1 Hz. Finally, (**d**) shows a screenshot of the ground station visualization during the simulations.

The detailed results for UAV 3 are depicted in Figure 18. It is shown how the sub-area is fully covered with the sensor on board, even considering a very slow data acquisition rate of 1 Hz.

## 7. Conclusions and Future Work

This paper has presented an algorithmic approach that allows one to tackle in a common framework the problems of area decomposition, partition and coverage in a multi-UAV remote sensing context. The produced mesh and associated graph manage to be consistent with the area properties and the capabilities of the UAVs and their on-board sensors. Two novel algorithms have been proposed to solve deadlock scenarios that can be usually found when performing area partition into sub-areas taking into account the relative capabilities of the UAVs.

The current framework is actually a generic waypoint planner that is consistent with the attributes and attitude of the on-board sensor. However, it does not take into consideration the UAV platform dynamics, even though the pitch and roll upper boundary turn rates are used to calculate the maximum triangle cell. Nevertheless, a platform might, in the case of a multirotor, or might not, in the case of a fast moving fixed wing, be able to follow sharp turns that are produced. Hence, this solution does not account for waypoint to waypoint flight trajectories. These issues are usually addressed by the flight controller, for example, by assuming that a waypoint has been visited if the UAV passes close by. This metric is task specific and, in real-world applications, user defined.

Regarding future work, a comparative study has to be performed regarding mesh generation optimization using Lloyd’s algorithm. In addition, removing vertices that the UAS has not managed to visit and replacing them online with the current position has to be tested in order to get information on the computational time versus the optimality of the trajectory analysis. On the other hand, uncertainties in the perception of the environment will be encoded in each of the graph vertices for the on-board computation of the trajectory, compensating for changes in the scenario and tasks. Finally, our final goal is to develop a complete system architecture for a team of heterogeneous UAS that will be able to perform in complex coastal areas, having minimal supervision during real flights.

## Figures and Tables

**Table 1:** An even distribution of initial locations for three and five UAVs, with different relative capabilities.

		FoV (15 m)		FoV (30 m)	
	#UAVs	moveRWS	moveAWP	moveRWS	moveAWP
**Metric *F* (m)**	3	333,861.84	333,909.67	82,768.44	84,979.76
**Metric *F* (m)**	5	437,988.74	439,642.85	129,879.24	131,516.96

**Table 2:** Random initial position distribution for three and five UAVs, with different relative capabilities. Like before, Algorithm 5 has performed better than Algorithm 4.

		FoV (15 m)		FoV (30 m)	
	#UAVs	moveRWS	moveAWP	moveRWS	moveAWP
**Metric *F* (m)**	3	508,801.74	508,751.513	211,395	214,945.82
**Metric *F* (m)**	5	566,971.55	568,819.45	151,389.621	155,269.99

**Table 3:** For each UAV, the difference from its given capability is shown after the initial baseline algorithm and after the moveRWS deadlock treatment algorithm. An average for all UAVs, as well as the total difference is shown below each experimental setup, where *G* is the metric defined in Equation 2 and area(R) is the area in $m^{2}$ of the whole region *R*. The UAVs have evenly distributed initial positions. Setups for 3, 4, 5 and 6 UAVs have been tested, with different relative capabilities and FoV values. Different Lloyd iterations on the mesh have been tested, ranging between 20 and 60.

UAV Capability %	FoV (15 m)			FoV (30 m)		
	Lloyd Iterations			Lloyd Iterations		
	20	30	60	20	30	60
**50%**	0.5/0.92	0/0.62	0/0.92	0.35/0.3	0.35/0.18	0.05/0.3
**30%**	6.74/0.14	6.74/0.89	6.88/1.21	6.95/0.21	7.19/0.65	6.86/0.25
**20%**	0.01/1.04	0.01/0.26	0.01/0.28	0/0.49	0.03/0.82	0.05/0.54
**Average%**	2.41/0.69	2.25/0.59	2.29/0.80	2.43/0.32	2.52/0.55	2.32/0.36
G/area(R)**%**	7.25/2.1	6.75/1.78	6.89/2.42	7.3/0.99	7.57/1.66	6.96/1.09
**20%**	0.02/0.02	0.02/0.08	0.02/0.06	0/0.41	0.05/0.12	0.05/0.45
**40%**	0/0.06	0/0.11	0/0.05	3.89/0.28	3.91/0.59	3.6/0.49
**20%**	0.96/0	1.11/0	0.78/0.06	0.48/0.17	0.48/0	0.62/0.12
**20%**	0/0.02	0/0.17	0/0.03	0/0.29	0/0.48	0/0.07
**Average%**	0.25/0.03	0.28/0.09	0.2/0.05	1.09/0.37	1.11/0.3	1.07/0.28
G/area(R)**%**	0.98/0.12	1.13/0.35	0.8/0.2	4.37/1.11	4.44/1.18	4.27/1.13
**20%**	0.15/0.38	0.15/0.6	0.18/0.01	0.15/0.11	0.11/0.59	0.11/1.36
**30%**	13.73/0.34	13.75/0.31	13.72/0.15	13.1/0.24	12.98/0.76	12.63/1.64
**20%**	0/0.31	0/0.42	0/0.08	0/1.6	0.3/0.81	0/0.66
**10%**	0/0.41	0/0.86	0/0.41	0/0.71	0/0.3	0/1.21
**20%**	0.21/0.65	0.05/0.14	0.21/0.36	0/1.01	0/0.36	0/1.61
**Average%**	2.81/0.42	2.79/0.47	2.28/0.35	2.65/0.92	2.68/0.56	2.55/0.9
G/area(R)**%**	14.09/2.1	13.95/2.33	14.11/1.01	13.25/3.69	13.39/2.81	12.74/4.5
**10%**	0.15/0.21	0.15/0.73	0.15/0.41	0.11/0.72	0/0.18	0.11/0.29
**20%**	0.3/1.04	0.3/1.37	0.3/1.35	0/1.83	0/1.37	0/0.43
**10%**	0.15/0.23	0.15/0.16	0.15/0.23	0.11/0.14	0/0.97	0.11/0.39
**30%**	11.26/0.38	11.45/0.69	11.2/0.53	11.94/0.84	12.64/0.83	10.75/0.96
**20%**	0.3/0.51	0.3/0.36	0.3/0.33	0.24/0.8	0/0.98	0.24/0.55
**10%**	0.15/0.19	0.15/0.47	0.15/0.15	0.11/1.04	0.11/1.25	0.11/0.98
**Average%**	2.05/0.41	2.08/0.63	2.04/0.5	2.09/0.9	2.13/0.93	1.89/0.6
G/area(R)**%**	12.32/2.47	12.5/3.78	12.25/3.01	12.51/5.36	12.75/5.58	11.32/3.59

**Table 4:** For each UAV, the difference from its given capability is shown after the initial baseline algorithm and after the moveRWS deadlock treatment algorithm. An average for all UAVs, as well as the total difference is shown below each experimental setup, where *G* is the metric defined in Equation 2 and area(R) is the area in $m^{2}$ of the whole region *R*. The UAVs have randomly distributed initial positions. Setups for 3, 4, 5 and 6 UAVs have been tested, with different relative capabilities and FoV values. Different Lloyd iterations on the mesh have been tested, ranging between 20 and 60.

UAV Capability %	FoV (15 m)			FoV (30 m)		
	Lloyd Iterations			Lloyd Iterations		
	20	30	60	20	30	60
**50%**	0/0.1	0/0	30.8/0.27	0/0	0/1.49	29.81/0.5
**30%**	1.19/0.02	0.08/0.02	0.01/0.16	0.024/0.024	20.9/0.65	0.01/0.57
**20%**	0.01/0.09	0.01/0.01	0.01/0.11	0.024/0.024	0.024/0.84	0.05/1.1
**Average%**	0.4/0.07	0.03/0.01	10.27/0.18	0.02/0.02	6.97/0.99	9.95/0.72
G/area(R)**%**	1.2/0.21	0.09/0.03	30.82/0.54	0.05/0.05	20.92/2.98	29.87/2.17
**20%**	0/0.43	15.37/0.22	2.19/0.49	0/0.730	14.2/1.66	0.62/0.05
**40%**	13.1/0.33	0/0.53	0/0.22	14/0.43	0/1.89	0.07/0.14
**20%**	6.48/0.31	0/0.06	2.64/0.35	6.24/0.56	0/0.12	1.8/0.16
**20%**	0/0.42	0/0.69	0/0.35	0/0.26	0/0.12	0.02/0.07
**Average%**	4.9/0.37	3.84/0.38	1.21/0.36	5.06/0.5	3.55/0.95	0.67/0.11
G/area(R)**%**	19.58/1.48	15.37/1.5	4.83/1.43	20.24/1.98	14.2/3.79	2.67/0.42
**20%**	0.01/0.03	0.01/0.21	11.23/0.77	0/1.8	0.02/1.43	10.78/0.4
**30%**	0.53/0.02	12.85/0.24	0.02/0.68	0/0.27	13.45/0.42	0.02/1.4
**20%**	0.01/0.03	0.01/0.11	11.59/2.65	6.24/0.57	6.88/0.44	10.78/0.64
**10%**	0.01/0.07	0.01/0.19	3.382/1.19	0/0.46	0.04/0.21	3.97/0.04
**20%**	0.01/0.14	0.01/0.14	0.01/1.36	0/1.03	0.02/0.37	0.02/0.4
**Average%**	0.11/0.05	2.58/0.18	5.25/1.33	1.25/0.82	4.12/0.57	5.11/0.58
G/area(R)**%**	0.57/0.27	12.89/0.89	26.24/6.64	6.24/4.12	20.59/2.87	25.57/2.88
**10%**	0.02/0.46	4.36/0.05	4.2/0.26	0.04/0.36	0.04/0.18	0.04/0.09
**20%**	0.02/1.02	4.23/0.26	0.01/0.06	0.05/0.3	0.05/0.36	0.05/1.49
**10%**	0.02/0.42	0.01/0.47	0.01/0.01	0.04/1.2	0/0.41	0.04/0.25
**30%**	19.04/0.5	0.01/1.22	0.01/0.01	20.32/0.74	20/1.48	19.85/0.31
**20%**	0.01/0.9	0.02/0.04	0.01/0.02	10.32/0.49	10.32/0.24	10.08/0.65
**10%**	0.02/0.66	0.01/0.58	0.01/0.2	0.04/0.028	0/1.01	0.04/0.22
**Average%**	3.18/0.66	1.44/0.43	0.71/0.09	5.13/0.52	5.07/0.61	5.02/0.5
G/area(R)**%**	19.13/3.96	8.64/2.62	4.25/0.56	30.81/3.12	30.41/3.68	30.1/3.01

**Table 5:** Values of the parameters considered for the UAVs in the simulations. The FoV projection size is the maximum cell side size of the triangulation. Angle γ is the on-board sensor pitch angle with respect to the horizontal plane. The relative capability percentages represent the capability of each UAV related to the whole area for covering purposes.

UAV	FoV Projection Size (m)	γ (deg)	Altitude (m)	Relative Capability
UAV 1	30	−45	100	20%
UAV 2	40	−45	80	30%
UAV 3	55	−45	120	50%

## Abbreviations

The following abbreviations are used in this manuscript:

UAV	Unmanned Aerial Vehicle
FoV	Field of View
CDT	Constrained Delaunay Triangulation
AWP	Antagonizing Wavefront Propagation
RWS	Reverse Watershed Schema
DLH	Deadlock Handling
ROS	Robotic Operating System
SITL	Software In The Loop
